# The role of nitric oxide in the dorsomedial periaqueductal gray (dmPAG) column in cardiovascular responses in urethane‐anesthetized male rats

**DOI:** 10.1002/ame2.12292

**Published:** 2022-11-22

**Authors:** Mohammad Najaftomaraei, Atiyeh Ghorbani, Alireza Rahimi, Reza Mohebbati, Sogol Sherkat, Mohammad Naser Shafei

**Affiliations:** ^1^ Department of Physiology, Faculty of Medicine Mashhad University of Medical Sciences Mashhad Iran; ^2^ Material Science and Metallurgy Engineering Islamic Azad University ‐ Karaj Branch Karaj Iran; ^3^ Department of Physiology, Faculty of Medicine Gonabad University of Medical Sciences Gonabad Iran; ^4^ Applied Biomedical Research Center Mashhad University of Medical Sciences Mashhad Iran; ^5^ Department of Physiology, School of Medicine Sabzevar University of Medical Sciences Sabzevar Iran; ^6^ Division of Neurocognitive Sciences, Psychiatry and Behavioral Sciences Research Center Mashhad University of Medical Sciences Mashhad Iran

**Keywords:** blood pressure, dorsomedial periaqueductal gray, L‐arginine, L‐NAME, nitric oxide

## Abstract

**Background:**

The dorsomedial periaqueductal gray (dmPAG) is a mesencephalic area and has numerous functions including cardiovascular regulation. Because nitric oxide (NO) is present in the dmPAG, here we investigate, the probable cardiovascular effect of NO in the dmPAG.

**Methods:**

Five groups (*n* = 6 for each group) were used as follows: (1) control; (2) L‐NAME (*N* ^G^‐nitro‐L‐arginine methyl ester, a NO synthase inhibitor, 90 nmol); (3) L‐arginine (L‐Arg, a precursor for NO, 60 nmol); (4) Sodium nitroprusside (SNP, a NO donor, 27 nmol); and (5) L‐Arg + L‐NAME. The cardiovascular parameters were recorded by a Power Lab device after cannulation of the femoral artery. Drugs were injected using a stereotaxic instrument. The changes (∆) in systolic blood pressure (SBP), mean arterial pressure (MAP), and heart rate (HR) were calculated at different times and compared to the control group.

**Results:**

Microinjection of L‐NAME significantly increased ∆SBP, ∆MAP, and ∆HR more than saline (from *p* < 0.05 to *p* < 0.001). L‐Arg only significantly increased ∆HR (*p* < 0.05). In the L‐Arg + L‐NAME group, the above parameters also significantly increased (from *p* < 0.01 to *p* < 0.05) but not as significantly as with L‐NAME alone. Microinjection of SNP significantly decreased ∆SBP and ∆MAP more than in the control and L‐NAME groups (from *p* < 0.01 to *p* < 0.001), but ∆HR did not change significantly.

**Conclusion:**

The results indicated that NO in dmPAG has an inhibitory effect on cardiovascular responses in anesthetized rats.

## Graphical abstract

In the current study, after anesthetized rat with urethan the femoral artery was cannulated, then its head was mounted on a stereotaxic instrument and drugs (saline‐NAME, L‐Arg, and L_Arg+ L‐NAME) microinjected into the dmPAG and cardiovascular responses that recorded by a Power lab were evaluated. We found an inhibitory cardiovascular effect of NO in dmPAG.

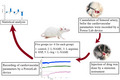

## 1 INTRODUCTION

The periaqueductal gray column (PAG) is a mesencephalic region involved in several functions including cardiovascular changes integrated with emotional behaviors.^1^ The PAG is divided into four distinct columns based on its functions, named lateral (lPAG), dorsolateral (dlPAG), ventrolateral (vlPAG), and dorsomedial (dmPAG).

The dmPAG column has been assoicaited with effects on the defensive (freeze, flight, or fight threat response) and aversive control of behaviors (inhibiting switch or off fear response). It has been reported that chemical or electrical incitement of dmPAG stimulates freezing, arousal, and escape reactions in animals.^2^ In addition, the dmPAG region is a major contributor to fear and anxiety mediation and associated defense mechanisms.^3^, ^4^ In the case of cardiovascular regulation, it has been shown that the dmPAG receives inputs from various divisions in ventrolateral medulla and spinal cord^5^ and develops outputs to the rostral ventrolateral medulla (RVLM, an important area in the control of the cardiovascular system), parabrachial complex (PB), and midline medulla (MM) as sources of premotor neurons.^5^ The PAG also receives excitatory inputs from higher regions such as dorsomedial hypothalamus (DMH).^6^

Various effects of this column on the cardiovascular system have been reported. For instance, microinjection of lipopolysaccharides (LPS) into the dmPAG causes a decrease in blood pressure (BP), and a non‐significant increase in heart rate (HR).^7^ In another study, microinjection of noradrenaline into this area evoked pressor responses and associated bradycardia.^8^

Nitric oxide (NO) is an important agent with several peripheral and central effects. Clinical trials have shown an association between hypertension, atherosclerosis, and hypoxia and the nitric oxide system.^9^ In the brain, this substance acts as a neurotransmitter and contributes to numerous functions including cardiovascular modulation. NO is synthesized by a family of enzymes, nitric oxide synthase (NOS) (Tai, 2007), with three different isoforms – endothelial (eNOS), inducible (iNOS), and neural (nNOS). L‐Arginine (L‐Arg) is a biological precursor of NO that via the effect of NOS produces citrulline and NO agents.^10^ NOS acts to regulate the cardiovascular system within the RVLM.^11^ In the peripheral system, it has been documented that NO plays an active role in the maintenance and regulation of vascular tone in different tissues, mediated by nonadrenergic noncholinergic (NANC) nerves^12^, ^13^ and aided by cholinergic stimulating agents.^13^ NOS isoforms are also immunohistochemically localized to distinct neuronal populations all over the brain.^14^ NO additionally produces a reduction in the outflow of the central sympathetic mechanism,^15^ as well as in BP and HR, in response to L‐glutamate intervention and baroreflex‐like control in the nucleus tractus solitarius (NTS).^16^ Other studies have shown the role of NO within the dPAG in modulating cardiovascular responses.^17^, ^18^ Blood pressure is significantly decreased by the administration of NO into the RVLM, and increased by the inhibition of NOS.^19^, ^20^ In a more recent study of electrophysiology, NO has been found to be a key factor in moderating the inhibitory effects of 5‐HT in the PAG system.^21^ In work conducted by our team, microinjection of NO into the dlPAG resulted in increased BP but decreased HR.^10^ NO synthase‐positive neurons were also identified in other parts of the brain such as paraventricular nucleus of the hypothalamus (PVN) and modulate the autonomic and endocrine effects on the cardiovascular activities.^22^ In addition, NO donor microinjection into the PVN reduced renal sympathetic nerve discharge (SND), mean arterial pressure (MAP),^6^ and HR in anesthetized rats,^7^, ^23^ but microinjection of NOS blockers stimulated excitatory cardiovascular responses.^23^, ^24^

Because NO has been shown to be present in this column of the PAG,^25^, ^26^ this study investigated the effect of NO microinjected into the dmPAG on the cardiovascular system, to reveal the way dmPAG relates to different control centers of the cardiovascular system in the brain and the relationship of NO with this system.

## 2 METHODS

### 2.1 Animals

In this experiment, 35 male Wistar rats weighing 230–250 g were used. Animals were housed individually in plastic cages in a temperature‐controlled room (25.8°C) and 12:12‐h light–dark cycle and had free access to water and food. Experimental procedures were carried out following protocols approved by the ethical review committee of the faculty of Mashhad University of Medical Sciences. (IR.MUMS.MEDICAL.REC.1399.314).

### 2.2 Drugs

The drugs used in this study were urethane (for anesthesia), L‐Arg (a NO precursor), *N* ^G^‐nitro‐L‐arginine methyl ester (L‐NAME, a nitric oxide synthase inhibitor), and sodium nitroprusside (SNP, a NO donor). All drugs used in this experiment were procured from Sigma, USA.

### 2.3 Experiment groups

Rats randomly were divided into five groups, as follows (*n* = 7 for each group):

1. Control group: microinjection of saline into the dmPAG.
2. L‐NAME: microinjection of 90 nmol L‐NAME into the dmPAG.^27^
3. L‐Arg, microinjection of 60 nmol L‐Arg into the dmPAG.^28^
4. SNP, microinjection of 27 nmol SNP into the dmPAG.
5. L‐Arg + L‐NAME: microinjection of 90 nmol L‐NAME, followed after 5 min by of 60 nmol L‐Arg.^29^

### 2.4 Surgical preparation

Rats were anesthetized with urethane (1.5 g/kg, IP). The left femoral artery was exposed and a 22‐gauge angiocatheter cannula (Indian Co.) filled with heparinized saline inserted into this artery. An angiocatheter was attached via a blood pressure transducer to a PowerLab (ID Instruments Co.)^30^ to monitor and continuously record mean arterial pressure (MAP),^6^ systolic blood pressure (SBP), and HR.^7^ For microinjection of drugs, the rats' heads were placed in a stereotaxic apparatus and fixed (Stoelting). The position of the dmPAG on the skull was determined according to the Paxinos rat brain atlas (AP: 0 mm; L: ±1.4 and H: 4.9 mm)^31^ and a hole was then drilled above the dmPAG column. All drugs (saline, L‐Arg, L‐NAME, L‐NAME+L‐Arg, and SNP) were microinjected into the dmPAG via a single‐barreled micropipette after stabilization of cardiovascular parameters. Injection was done manually via a PE‐10 tube attached to the barreled micropipettes. To inject the drugs, the screw of the injector was slowly rotated so that the drugs enter the nucleus slowly (over about 30 s). After drug injection, the resulting responses were recorded after 20 min and the peak change (∆) was calculated.

### 2.5 Statistical analysis

The changes (∆) in cardiovascular parameters (MAP, SBP and HR) were calculated and were expressed as means ± SEM. The time course of the changes in the parameters were statistically analyzed using repeated measures ANOVA, while maximal changes were analyzed using one‐way ANOVA, with Tukey's post hoc test. *p* < 0.05 was assumed to be statistically significant.

## 3 RESULTS

### 3.1 Cardiovascular responses to microinjection of saline into the dmPAG

After the stabilizing period, saline was microinjected into the dmPAG. The results indicated that saline did not significantly alter SBP, MAP, and HR compared to pre‐injection (*p* > 0.05).

### 3.2 Cardiovascular responses to microinjection of L‐NAME and L‐Arg into the dmPAG

**FIGURE 1:**
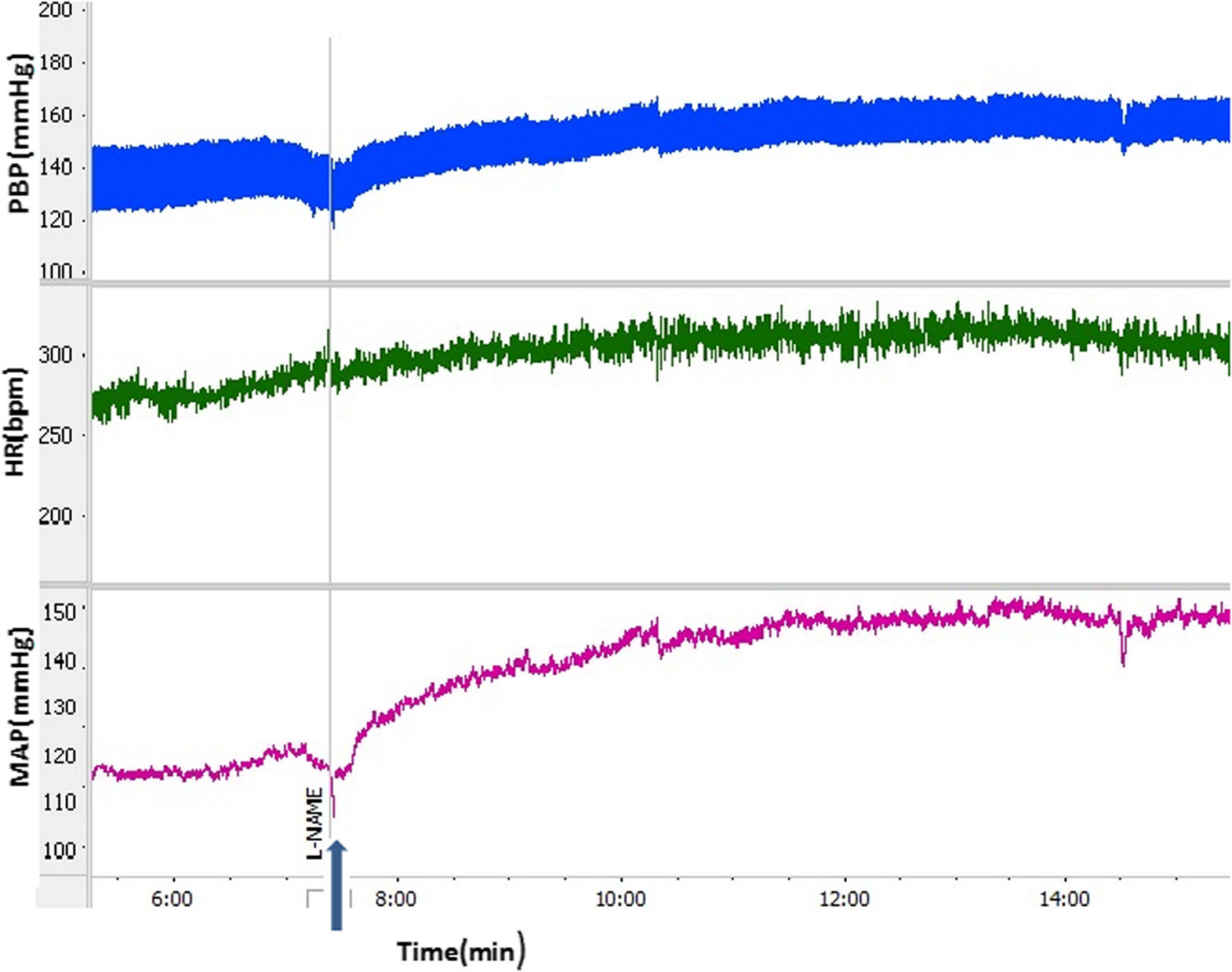
Sample recordings after microinjection of L‐NAME into the dmPAG. HR, heart rate; MAP, mean arterial pressure; PBP, pulsative blood pressure.

**FIGURE 2:**
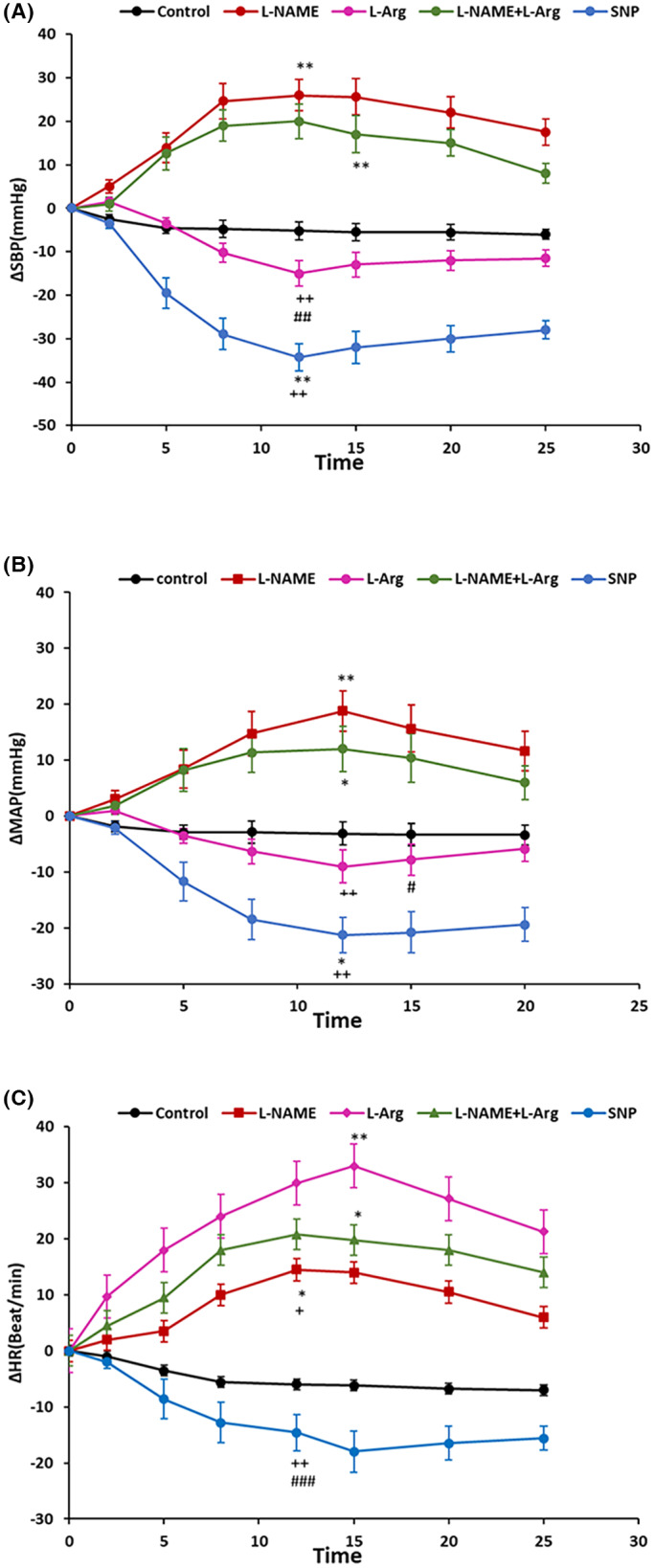
Time course of ∆SBP (A), ∆MAP (B), and ∆HR (C), after microinjection of saline, L‐NAME, L‐Arg, L‐NAME+L‐Arg, and SNP into the dmPAG. L‐NAME: *N* ^G^‐nitro‐L‐arginine methyl ester; a NOS inhibitor, L‐Arg: L‐arginine, a NO precursor, SNP: sodium nitroprusside, a NO donor. Statistical analysis: repeated measures ANOVA; *n* = 7. *p < 0.05; **p < 0.01; ***p < 0.001 compared to control. ^+^ *p* < 0.05; ^++^ *p* < 0.01; ^+++^ *p* < 0.001 compared to L‐NAME. ^#^ *p* < 0.05; ^##^ *p* < 0.01; ^###^ *p* < 0.001 compared to L‐NAME+L‐Arg.

**FIGURE 3:**
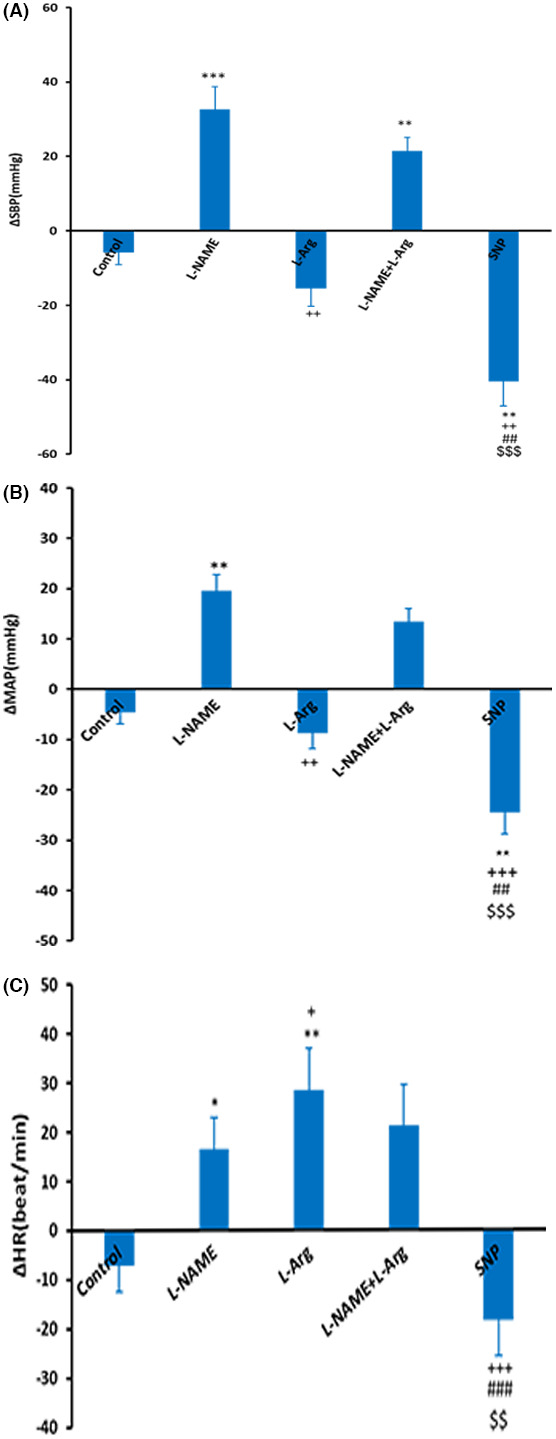
Peak changes in cardiovascular responses after microinjection of the L‐NAME, L‐Arg, L‐Arg + L‐NAME, and SNP into the dmPAG. Statistical analysis: one‐way ANOVA followed by Tukey's post hoc test, *n* = 7. *p < 0.05; **p < 0.01; ***p < 0.001 compared to control. ^+^ *p* < 0.05; ^++^ *p* < 0.01; ^+++^ *p* < 0.001 compared to L‐NAME. ^#^ *p* < 0.05; ^##^ *p* < 0.01; ^###^ *p* < 0.001 compared to L‐NAME+L‐Arg.

**FIGURE 4:**
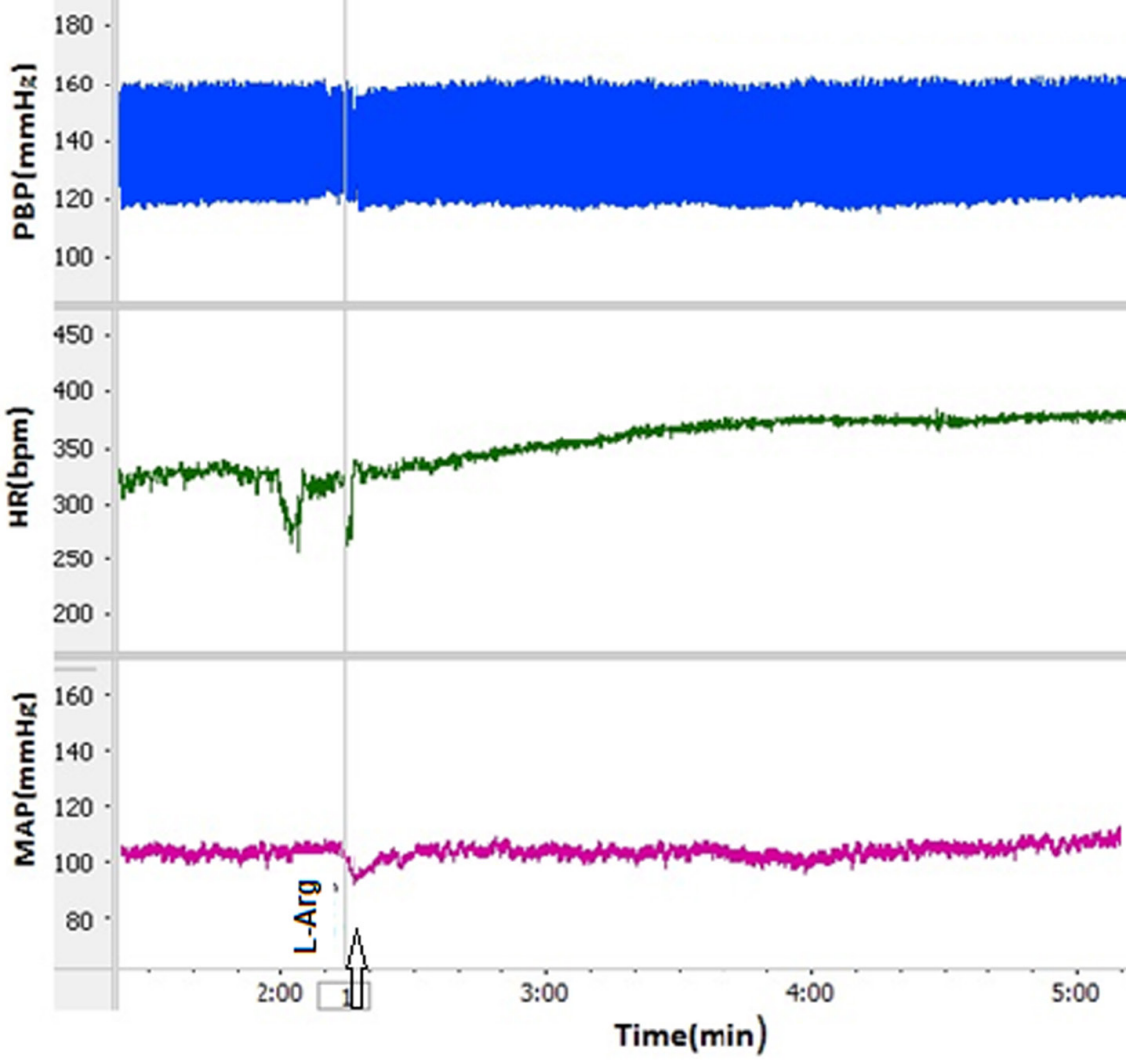
Sample recordings after microinjection of L‐arginine into the dmPAG. HR, heart rate; MAP, mean arterial pressure; PBP, pulsative blood pressure.

Microinjection of L‐NAME into the dmPAG increased ΔSBP, ΔMAP, and ΔHR compared to the control group (Figure 1). The time courses of the changes in the responses are shown in Figure 2. ΔSBP, ΔMAP and ΔHR increased with respect to saline over time (from *p* < 0.05 to *p* < 0.001). Peak change results also showed that ΔSBP (32.6 ± 6.2 mmHg vs. −5.8 ± 3.3 mmHg for saline; *p* < 0.001), ΔMAP (19.5 ± 3.3 mmHg vs. −4.6 ± 2.3 mmHg for saline; *p* < 0.01), and ΔHR (16.5 ± 6.4 beats/min vs. −7.2 ± 5.3 beats/min for saline; *p* < 0.05; Figure 3) were significantly greater than in the control group (*p* < 0.05 to *p* < 0.001). Microinjection of L‐Arg decreased SBP and MAP and increased HR (Figure 4). Time course changes after microinjection of L‐Arg also indicated that ΔSBP and ΔMAP decreased non‐significantly compared to the control group over time (Figure 2A,B). Peak changes in ΔSBP (−15.5 ± 4.7 mmHg vs. 5.8 ± 3.3 mmHg for saline) and ΔMAP (−8.8 ± 3 mmHg vs. −4.6 ± 2.3 mmHg for saline) were not significantly different to peak changes in the control group (Figure 3A,B). However, the time course and peak changes of ΔHR significantly increased with respect to the control group (28.32 ± 8.6 beats/min vs. −7.2 ± 5.3 beats/min for saline; *p* < 0.01, Figures 2C and 3C). The time course and peak changes of ΔSBP and ΔMAP in the L‐Arg group were significantly lower compared to the L‐NAME group: ΔSBP(−15.5 ± 4.7 mmHg vs. 32.6 ± 6.2 mmHg for L‐NAME; *p* < 0.001) and ΔMAP (−8.8 ± 3 mmHg vs. 19.5 ± 3.3 mmHg for L‐NAME; *p* < 0.01). ΔHR increased in both the L‐NAME and L‐Arg groups but the effect of L‐Arg was significantly higher than that of L‐NAME (L‐Arg: 28.32 ± 8.6 beats/min vs. L‐NAME: 16.5 ± 6.4 beats/min; *p* < 0.05).

### 3.3 Cardiovascular responses to co‐injection of L‐NAME+L‐Arg into the dmPAG

**FIGURE 5:**
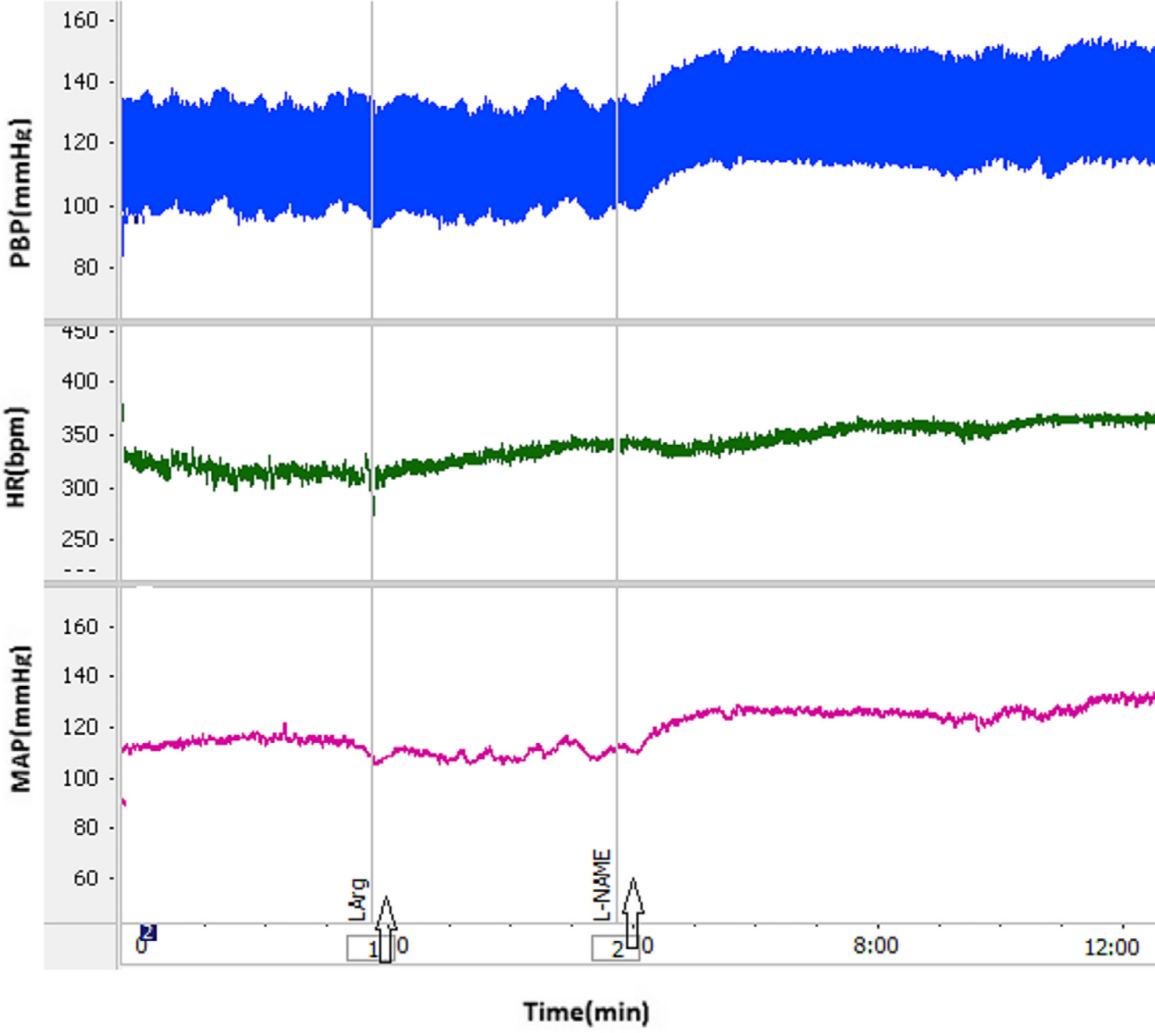
Sample recordings after co‐injection of L‐NAME+L‐Arg into the dmPAG. HR, heart rate; MAP, mean arterial pressure; PBP, pulsative blood pressure.

Co‐injection of L‐Arg and L‐NAME (L‐NAME+L‐Arg) into the dmPAG increased SBP, MAP and HR (Figure 5). ΔSBP, ΔMAP and ΔHR in the L‐NAME+L‐Arg group were significantly higher than in the control group over time (from *p* < 0.05 to *p* < 0.01, Figure 2A,B). Peak changes in ΔSBP, ΔMAP and ΔHR in the L‐NAME+L‐Arg group were also was significantly increased with respect to saline (ΔSBP: 21.5 ± 3.6 mmHg vs. saline: −5.8 ± 3.3 mmHg; *p* < 0.01; ΔMAP: 13.5 ± 2.6 mmHg vs. saline: −4.6 ± 2.3 mmHg; *p* < 0.05; ΔHR: 21.06 ± 8.4 beats/min vs. saline: −7.2 ± 5.3 beats/min; *p* < 0.05; Figure 3A,B). The cardiovascular changes in the L‐NAME+L‐Arg group were not significant compared to the L‐NAME group (ΔSBP: 21.5 ± 3.6 mmHg vs. L‐NAME: 32.6 ± 6.2 mmHg; ΔMAP: 13.5 ± 2.6 mmHg vs. L‐NAME: 19.5 ± 3.3 mmHg; ΔHR: 21.06 ± 8.4 beats/min vs. L‐NAME: 16.5 ± 6.4 beats/min) but were more significant than in the L‐Arg group.

### 3.4 Cardiovascular responses to microinjection of SNP into the dmPAG

**FIGURE 6:**
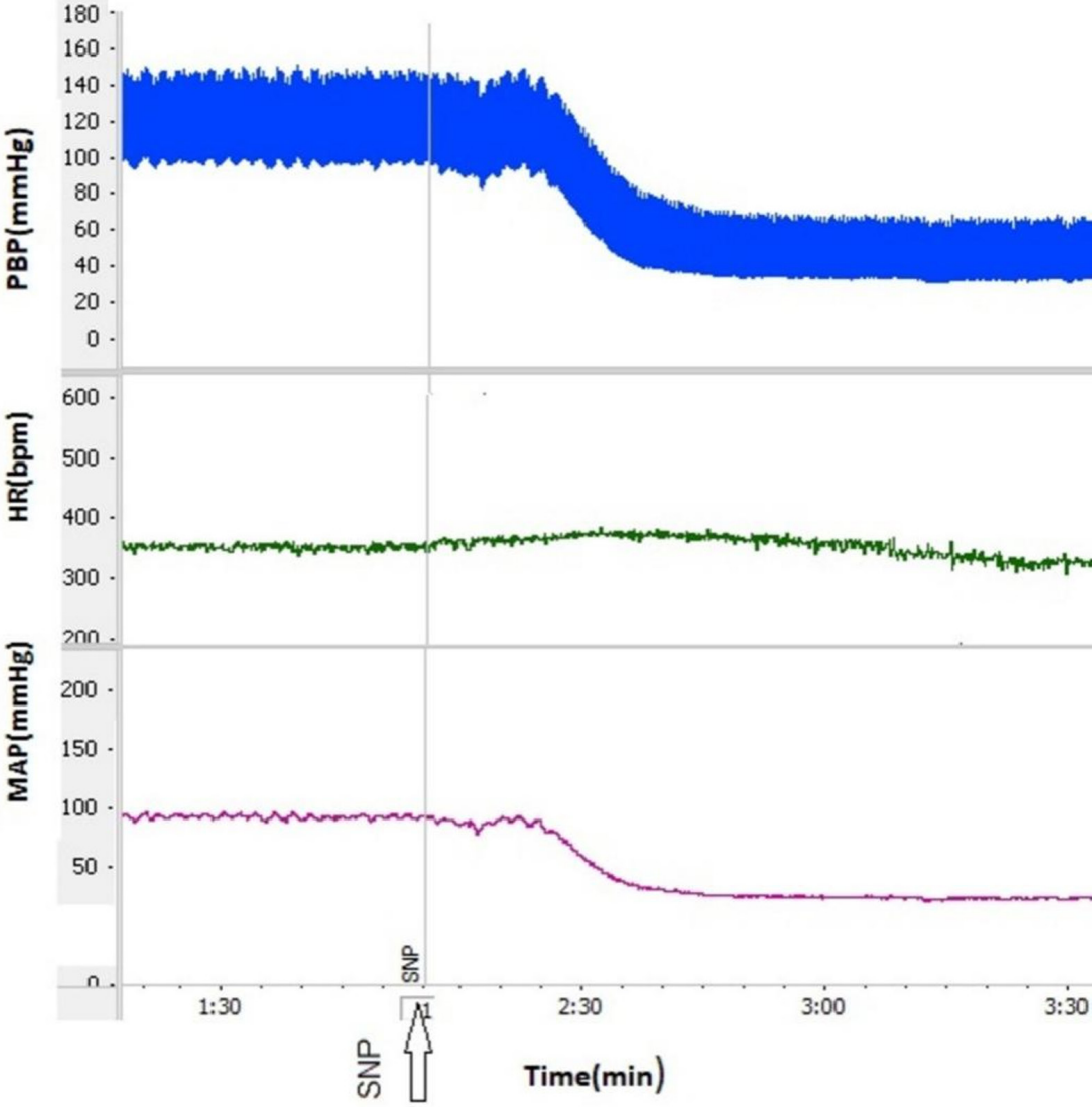
Sample recordings after microinjection of sodium nitroprusside (SNP) into the dmPAG. HR, heart rate; MAP, mean arterial pressure; PBP, pulsative blood pressure.

In this group, microinjection of SNP into the dmPAG decreased SBP and MAP while HR did not change (Figure 6). The time courses of the changes indicated that ΔSBP and ΔMAP significantly decreased compared to the control group over time (*p* < 0.01 Figure 2A,B). Peak changes in ΔSBP and ΔMAP in this group were also significantly lower than in the control (ΔSBP: −40.52 ± 6.54 mmHg vs. saline: −5.8 ± 3.3 mmHg; *p* < 0.01; ΔMAP: −24.5 ± 4.32 mmHg vs. saline: −4.6 ± 2.3 mmHg; *p* < 0.01; Figure 3A,B). Time courses and peak changes of ΔHR decreased non‐significantly compared to saline (ΔHR: −18.54 ± 7.2 beats/min vs. saline: −7.2 ± 5.3 beats/min; Figures 2C and 3C). Time courses and peak changes of the cardiovascular parameters of the SNP group were also significant in comparison to the L‐NAME, L‐Arg and L‐NAME+L‐Arg groups (Figures 2 and 3).

## 4 DISCUSSION

This experimental research evaluated the cardiovascular effects of the nitrergic system of dmPAG in intact rats. Our results showed that microinjection of L‐NAME increases all cardiovascular parameters. Co‐injection L‐NAME and L‐Arg (L‐NAME+L‐Arg) also increased all these parameters. Injection of L‐Arg only significantly increased of HR, and SNP significantly decreased SBP and MAP but had no significant effect on HR. Based on these results, we showed that the dmPAG nitrergic system has an inhibitory impact on the central cardiovascular activity.

NO‐producing neurons exist in numerous areas of the brain including the PVN,^32^ NTS and RVLM areas^33^ that regulate cardiovascular function. There are, however, questions with regard to the role of NO in the central cardiovascular function. Some previous studies^34^, ^35^ showed that NO by decreasing sympathetic vasomotor neuron activity reduces blood pressure,^35^ while other studies reported that NO could increase blood pressure^10^, ^36^ The mechanisms of these opposing effects are unknown. However, since NOS has 3 isoforms (eNOS, iNOS and nNOS), we speculate that the NO produced by each of these isoforms in the dmPAG has different effects on neurons involved in the cardiovascular activity. In line with this opinion, previous studies reported different cardiovascular effects of the NOS isoforms in RVLM^37^, ^38^. Therefore, we propose that the cardiovascular effects of the three NOS isoforms should be investigated separately.

Projections from the PAG to brain areas involved in cardiovascular regulation, including RVLM,^39^ have been reported. The sympathetic preganglionic neurons (SPNs) located in the spinal cord are also mainly driven by RVLM premotor neurons and have a great impact upon cardiovascular regulation.^40^ In addition, microinjection of L‐Arg into RVLM was shown to decrease BP^41^, ^42^ while L‐NAME microinjection caused BP and HR elevations,^43^, ^44^ providing evidence for an inhibitory effect of NO in the RVLM. Therefore, we suggest that NO has the potential to participate in cardiovascular regulation by modulation of the dmPAG–RVLM–IML pathway. In addition, cardiovascular responses were decreased by microinjection of SNP into the PVN and PPT areas, whereas these responses were observed to increase following L‐NAME microinjection into these areas.^28^, ^29^ Due to the connection between the PVN and PPT areas and dmPAG, we suggest that the presence of NO in dmPAG affects cardiovascular function these areas. However, future experimental research is needed to confirm this hypothesis. In addition, it has been claimed that sympathetic activity is modulated by the bidirectional connection of PAG to the hypothalamus, specially DMH,^39^, ^45^, ^46^, ^47^ and hence alterations in cardiovascular activity by NO may be innervated by indirect pathways such as the dmPAG–DMH–RVLM‐spinal cord pathway.

It has also been shown that caudal raphe nuclei receive afferents preferentially from the dmPAG^48^ and also that projections from dmPAG to the lateral parabrachial nucleus could mediate cardiorespiratory changes evoked by the dmPAG.^49^, ^50^ Therefore, it is possible that the nitrergic system of the dmPAG via these areas could result in cardiovascular regulation. The PAG innervates regions surrounded by the nucleus ambiguous^2^ and dorsal motor nucleus of the vagus (DMV) containing cardio vagal neurons.^51^, ^52^, ^53^

NO has been found to interact with some other neurotransmitters such as acetylcholine,^20^ glutamate, and gamma‐aminobutyric acid (GABA) in several areas of the brain.^54^, ^55^, ^56^ Since glutamate in the dmPAG has been observed to have a cardiovascular effect,^57^ its interaction with NO may inhibit cardiovascular effects in the dmPAG. Such an effect has been reported by Ishide et al,^58^ who demonstrated an interaction between NO and glutamate in the RVLM. Similarly, it is possible that NO and glutamate affect each other in the dmPAG.

This study revealed that L‐NAME significantly increased SBP, MAP, and HR parameters. It was shown that L‐NAME, an L‐Arg analog, competitively binds to NO synthase (Tai, 2007), attenuating NO production and its metabolic pathway.^59^ When NOS is inhibited, NO cannot be synthesized, decreasing L‐Arg and NO bioavailability in the dmPAG and thereby increasing cardiovascular parameters. To confirm this claim, L‐NAME was co‐injected with L‐Arg in another group. The results suggest that there was no significant cardiovascular effect of L‐NAME and L‐NAME+L‐Arg and the cardiovascular activity was likely modulated by the NO synthesized in dmPAG. More studies should be done to gain a clear picture of NO production mechanisms and effects in the dmPAG.

In this study, injection of L‐Arg into the dmPAG led to a significant rise in HR and no significant change in BP. Although the production of NO could be elicited by L‐Arg, the exact mechanism is still unknown. Due to its low production, NO does not produce a high inhibitory effect on BP. It also has an inhibitory effect on both sympathetic and parasympathetic activities.^60^

In addition to inhibition of the sympathetic system, it seems that NO imposes an inhibitory effect on the parasympathetic activity, which raises HR. Another possible cause could be the result of the effect of L‐Arg on baroreceptor reflexes. However, defining the mechanism is complicated and requires further investigation. In this study, it was observed that in the dmPAG region the cardiovascular effect of L‐Arg produces NO through the enzyme NOS. This is different from the direct effect of SNP on NO production. The mechanism for this dual effect of NO is not yet known despite demonstration of varying effects at a diverse range of doses and concentrations in previous studies. It is also possible that, unlike L‐Arg which induces a small amount of NO production by NOS, with a minor effect on BP, SNP releases more NO, with major effects on both BP and HR. In addition, our study results suggest that at lower doses NO raises HR, yet its effect is inhibitory on HR at higher doses. Our results support previous findings that the effects of NO on cardiovascular parameters vary with different administered doses. Further studies should to be conducted on NO transmission pathways to understand the mechanisms of these differing effects of NO on BP in different areas of the brain.

In sum, the results of this study demonstrate that NO has cardiovascular effects which are mainly inhibitory. The study also demonstrates that the production of NO following injection of L‐arginine (a NO precursor) is different from that of SNP (direct NO release), and therefore the different effects of L‐Arg and SNP may be the result of the different concentrations of NO produced.

## AUTHOR CONTRIBUTIONS

Ghorbani A, Najaf Tomara M; performed methodology, investigation and formal analysis. Ghorbani A, Najaf Tomara M, Mohebbati R, Rahimi R, and Sherkat S; Performed writing and manuscript preparation. Shafei MN performed study conception and supervision.

## CONFLICT OF INTEREST

The authors declare that they have no conflict of interest.

## References

[1] Carrive P . The periaqueductal gray and defensive behavior: functional representation and neuronal organization. Behav Brain Res. 1993;58:27‐47. 8136048 10.1016/0166-4328(93)90088-8

[2] Borelli KG , Brandão ML . Effects of ovine CRF injections into the dorsomedial, dorsolateral and lateral columns of the periaqueductal gray: a functional role for the dorsomedial column. Horm Behav. 2008;53:40‐50. 17920596 10.1016/j.yhbeh.2007.08.013

[3] Carrive P , Bandler R , Dampney R . Anatomical evidence that hypertension associated with the defence reaction in the cat is mediated by a direct projection from a restricted portion of the midbrain periaqueductal grey to the subretrofacial nucleus of the medulla. Brain Res. 1988;460:339‐345. 2465061 10.1016/0006-8993(88)90378-2

[4] Bandler R , Depaulis A . Midbrain Periaqueductal Gray Control of Defensive Behavior in the Cat and the Rat. The Midbrain Periaqueductal Gray Matter. Springer; 1991:175‐198.

[5] Dampney RA , Furlong TM , Horiuchi J , Iigaya K . Role of dorsolateral periaqueductal grey in the coordinated regulation of cardiovascular and respiratory function. Auton Neurosci. 2013;175:17‐25. 23336968 10.1016/j.autneu.2012.12.008

[6] Da Silva LG Jr , Menezes R , Villela DC , Fontes MA . Excitatory amino acid receptors in the periaqueductal gray mediate the cardiovascular response evoked by activation of dorsomedial hypothalamic neurons. Neuroscience. 2006;139:1129‐1139. 16458440 10.1016/j.neuroscience.2005.12.041

[7] Mohebbati R , Nejad Shahrokh Abadi R , Alikhani V , Shafei MN . The cardiovascular responses after lipopolysaccharide microinjection into the dorsomedial periaqueductal gray in rats. Physiol Pharmacol. 2021;25:334‐340.

[8] Pelosi GG , Corrêa FMA . Cardiovascular effects of noradrenaline microinjected into the dorsal periaqueductal gray area of unanaesthetized rats. Eur J Neurosci. 2005;22:3188‐3194. 16367785 10.1111/j.1460-9568.2005.04511.x

[9] Panza JA , Quyyumi AA , Brush JE Jr , Epstein SE . Abnormal endothelium‐dependent vascular relaxation in patients with essential hypertension. N Engl J Med. 1990;323:22‐27. 2355955 10.1056/NEJM199007053230105

[10] Nejadshahrokhabadi R , Zangouei AS , Mohebbati R , Shafei MN . Determining the cardiovascular effects of nitric oxide in the dorsolateral periaqueductal gray (dlPAG) in anaesthetised rats. J Taibah Univ Med Sci. 2020;15:502‐508. 33318742 10.1016/j.jtumed.2020.10.004 PMC7715464

[11] Lee YH , Tsai MC , Li TL , Dai YW , Huang SC , Hwang LL . Spontaneously hypertensive rats have more orexin neurons in the hypothalamus and enhanced orexinergic input and orexin 2 receptor‐associated nitric oxide signalling in the rostral ventrolateral medulla. Exp Physiol. 2015;100:993‐1007. 26096870 10.1113/EP085016

[12] Ignarro LJ , Bush PA , Buga GM , Wood KS , Fukuto JM , Rajfer J . Nitric oxide and cyclic GMP formation upon electrical field stimulation cause relaxation of corpus cavernosum smooth muscle. Biochem Biophys Res Commun. 1990;170:843‐850. 2166511 10.1016/0006-291x(90)92168-y

[13] Mcmahon T , Hood J , Kadowitz P . Pulmonary vasodilator response to vagal stimulation is blocked by N omega‐nitro‐L‐arginine methyl ester in the cat. Circ Res. 1992;70:364‐369. 1735136 10.1161/01.res.70.2.364

[14] Bredt DS , Hwang PM , Snyder SH . Localization of nitric oxide synthase indicating a neural role for nitric oxide. Nature. 1990;347:768‐770. 1700301 10.1038/347768a0

[15] Togashi H , Sakuma I , Yoshioka M , et al. A central nervous system action of nitric oxide in blood pressure regulation. J Pharmacol Exp Ther. 1992;262:343‐347. 1378094

[16] Di Paola ED , Vidal MJ , Nisticò G . L‐glutamate evokes the release of an endothelium‐derived relaxing factor‐like substance from the rat nucleus tractus solitarius. J Cardiovasc Pharmacol. 1991;17:S269‐S272.

[17] Reichling DB , Kwiat GC , Basbaum AI . Anatomy, physiology and pharmacology of the periaqueductal gray contribution to antinociceptive controls. Prog Brain Res. 1988;77:31‐46. 3064173 10.1016/s0079-6123(08)62777-6

[18] Karlsson GA , Chaitoff KA , Hossain S , Böhlke M , Maher TJ , Ally A . Modulation of cardiovascular responses and neurotransmission during peripheral nociception following nNOS antagonism within the periaqueductal gray. Brain Res. 2007;1143:150‐160. 17320064 10.1016/j.brainres.2007.01.101

[19] Kagiyama S , Tsuchihashi T , Abe I , Fujishima M . Cardiovascular effects of nitric oxide in the rostral ventrolateral medulla of rats. Brain Res. 1997;757:155‐158. 9200511 10.1016/s0006-8993(97)00336-3

[20] Zanzinger J , Czachurski J , Seller H . Inhibition of basal and reflex‐mediated sympathetic activity in the RVLM by nitric oxide. Am J Physiol‐Regul Integr Compar Physiol. 1995;268:R958‐R962. 10.1152/ajpregu.1995.268.4.R958 7733407

[21] Lovick T . Role of nitric oxide in medullary raphe‐evoked inhibition of neuronal activity in the periaqueductal gray matter. Neuroscience. 1996;75:1203‐1209. 8938753 10.1016/0306-4522(96)00325-9

[22] Li Y , Zhang W , Stern JE . Nitric oxide inhibits the firing activity of hypothalamic paraventricular neurons that innervate the medulla oblongata: role of GABA. Neuroscience. 2003;118:585‐601. 12710969 10.1016/s0306-4522(03)00042-3

[23] Zhang K , Patel KP . Effect of nitric oxide within the paraventricular nucleus on renal sympathetic nerve discharge: role of GABA. Am J Physiol‐Regul Integr Compar Physiol. 1998;275:R728‐R734. 10.1152/ajpregu.1998.275.3.R728 9728069

[24] Martins‐Pinge MC , Becker LK , MRL G , et al. Attenuated pressor responses to amino acids in the rostral ventrolateral medulla after swimming training in conscious rats. Auton Neurosci. 2005;122:21‐28. 16139573 10.1016/j.autneu.2005.07.007

[25] Xing J , Li D‐P , Li J . Role of GABA receptors in nitric oxide inhibition of dorsolateral periaqueductal gray neurons. Neuropharmacology. 2008;54:734‐744. 18222497 10.1016/j.neuropharm.2007.12.008 PMC2955627

[26] Ally A , Powell I , Ally MM , Chaitoff K , Nauli SM . Role of neuronal nitric oxide synthase on cardiovascular functions in physiological and pathophysiological states. Nitric Oxide. 2020;102:52‐73. 32590118 10.1016/j.niox.2020.06.004 PMC7375925

[27] Tai M‐H , Weng W‐T , Lo W‐C , et al. Role of nitric oxide in α‐melanocyte‐stimulating hormone‐induced hypotension in the nucleus tractus solitarii of the spontaneously hypertensive rats. J Pharmacol Exp Ther. 2007;321:455‐461. 17283224 10.1124/jpet.106.118299

[28] Shafei MN , Nikyar T , Hosseini M , Niazmand S , Paseban M . Cardiovascular effects of nitrergic system of the pedunculopontine tegmental nucleus in anesthetized rats. Iran J Basic Med Sci. 2017;20:776. 28852442 10.22038/IJBMS.2017.9009 PMC5569585

[29] Busnardo C , Crestani CC , Tavares RF , Resstel LB , Correa FM . Cardiovascular responses to L‐glutamate microinjection into the hypothalamic paraventricular nucleus are mediated by a local nitric oxide‐guanylate cyclase mechanism. Brain Res. 2010;1344:87‐95. 20478280 10.1016/j.brainres.2010.05.023

[30] Mohebbati R , Hosseini M , Khazaei M , Rad AK , Shafei MN . Involvement of the 5‐HT1A receptor of the cuneiform nucleus in the regulation of cardiovascular responses during normal and hemorrhagic conditions. Iran J Basic Med Sci. 2020;23:858. 32774806 10.22038/ijbms.2020.40453.9579 PMC7395185

[31] Paxinos G . Paxinos & Watson the Rat Brain in Stereotaxic Coordinates: The New Coronal Set. Elsevier; 2005.

[32] Miyagawa A , Okamura H , Ibata Y . Coexistence of oxytocin and NADPH‐diaphorase in magnocellular neurons of the paraventricular and the supraoptic nuclei of the rat hypothalamus. Neurosci Lett. 1994;171:13‐16. 8084473 10.1016/0304-3940(94)90592-4

[33] Dun N , Dun S , Förstermann U . Nitric oxide synthase immunoreactivity in rat pontine medullary neurons. Neuroscience. 1994;59:429‐445. 7516501 10.1016/0306-4522(94)90607-6

[34] Zhang K , Mayhan WG , Patel KP . Nitric oxide within the paraventricular nucleus mediates changes in renal sympathetic nerve activity. Am J Physiol‐Regul Integr Compar Physiol. 1997;273:R864‐R872. 10.1152/ajpregu.1997.273.3.R864 9321861

[35] Tseng C‐J , Liu H‐Y , Lin H‐C , Ger LP , Tung CS , Yen MH . Cardiovascular effects of nitric oxide in the brain stem nuclei of rats. Hypertension. 1996;27:36‐42. 8591885 10.1161/01.hyp.27.1.36

[36] Martins‐Pinge MC , Baraldi‐Passy I , Lopes OU . Excitatory effects of nitric oxide within the rostral ventrolateral medulla of freely moving rats. Hypertension. 1997;30:704‐707. 9323009 10.1161/01.hyp.30.3.704

[37] Chan J , Wang S‐H , Chan S . Differential roles of iNOS and nNOS at rostral ventrolateral medulla during experimental endotoxemia in the rat. Shock (Augusta, Ga). 2001;15:65‐72. 11198360 10.1097/00024382-200115010-00011

[38] Martins‐Pinge MC , Garcia MR , Zoccal DB , Crestani CC , Pinge‐Filho P . Differential influence of iNOS and nNOS inhibitors on rostral ventrolateral medullary mediated cardiovascular control in conscious rats. Auton Neurosci. 2007;131:65‐69. 16905370 10.1016/j.autneu.2006.07.004

[39] Farkas E , Jansen AS , Loewy AD . Periaqueductal gray matter input to cardiac‐related sympathetic premotor neurons. Brain Res. 1998;792:179‐192. 9593884 10.1016/s0006-8993(98)00029-8

[40] Guyenet PG . The sympathetic control of blood pressure. Nat Rev Neurosci. 2006;7:335‐346. 16760914 10.1038/nrn1902

[41] Shapoval L , Sagach V , Pobegailo L . Nitric oxide influences ventrolateral medullary mechanisms of vasomotor control in the cat. Neurosci Lett. 1991;132:47‐50. 1787918 10.1016/0304-3940(91)90430-2

[42] Chen SY , Mao SP , Chai CY . Role of nitric oxide on pressor mechanisms within the dorsomedial and rostral ventrolateral medulla in anaesthetized cats. Clin Exp Pharmacol Physiol. 2001;28:155‐163. 11207669 10.1046/j.1440-1681.2001.03434.x

[43] Chowdhary S , Townend JN . Role of nitric oxide in the regulation of cardiovascular autonomic control. Clin Sci. 1999;97:5‐17. 10369789

[44] Hirooka Y , Polson JW , Dampney R . Pressor and sympathoexcitatory effects of nitric oxide in the rostral ventrolateral medulla. J Hypertens. 1996;14:1317‐1324. 8934360 10.1097/00004872-199611000-00010

[45] Dampney R . The subretrofacial vasomotor nucleus: anatomical, chemical and pharmacological properties and role in cardiovascular regulation. Prog Neurobiol. 1994;42:197‐227. 8008825 10.1016/0301-0082(94)90064-7

[46] Moraes G , Mendonça M , Mourão A , et al. Ventromedial medullary pathway mediating cardiac responses evoked from periaqueductal gray. Auton Neurosci. 2020;228:102716. 32882606 10.1016/j.autneu.2020.102716

[47] Draberova L , Bugajev V , Potuckova L , et al. Transmembrane adaptor protein PAG/CBP is involved in both positive and negative regulation of mast cell signaling. Mol Cell Biol. 2014;34:4285‐4300. 25246632 10.1128/MCB.00983-14 PMC4248753

[48] Vianna D , Brandão M . Anatomical connections of the periaqueductal gray: specific neural substrates for different kinds of fear. Braz J Med Biol Res. 2003;36:557‐566. 12715074 10.1590/s0100-879x2003000500002

[49] Van Bockstaele EJ , Aston‐Jones G , Pieribone VA , Ennis M , Shipley MT . Subregions of the periaqueductal gray topographically innervate the rostral ventral medulla in the rat. J Comp Neurol. 1991;309:305‐327. 1717516 10.1002/cne.903090303

[50] Krout KE , Jansen AS , Loewy AD . Periaqueductal gray matter projection to the parabrachial nucleus in rat. J Comp Neurol. 1998;401:437‐454. 9826272

[51] Izzo P , Deuchars J , Spyer K . Localization of cardiac vagal preganglionic motoneurones in the rat: immunocytochemical evidence of synaptic inputs containing 5‐hydroxytryptamine. J Comp Neurol. 1993;327:572‐583. 8440781 10.1002/cne.903270408

[52] Hopkins DA , Bieger D , De Vente J , Steinbusch HW . Vagal efferent projections: viscerotopy, neurochemistry and effects of vagotomy. Prog Brain Res. 1996;107:79‐96. 8782514 10.1016/s0079-6123(08)61859-2

[53] Alikhani V , Nikyar T , Mohebbati R , Shafei MN , Ghorbani A . Cardiovascular responses induced by the activation of muscarinic receptors of the pedunculopontine tegmental nucleus in anesthetized rats. Clin Exp Hypertens. 2022;44:297‐305. 10.1080/10641963.2021.2007944 35266430

[54] Zanzinger J , Czachurski JR , Seller H . Neuronal nitric oxide reduces sympathetic excitability by modulation of central glutamate effects in pigs. Circ Res. 1997;80:565‐571. 9118488 10.1161/01.res.80.4.565

[55] Spyer K . Annual review prize lecture. Central nervous mechanisms contributing to cardiovascular control. J Physiol. 1994;474:1‐19. 8014887 10.1113/jphysiol.1994.sp019997 PMC1160290

[56] Gordon FJ . Aortic baroreceptor reflexes are mediated by NMDA receptors in caudal ventrolateral medulla. Am J Physiol‐Regul Integr Compar Physiol. 1987;252:R628‐R633. 10.1152/ajpregu.1987.252.3.R628 2881491

[57] Pelosi GG , Busnardo C , Tavares RF , Corrêa FM . Cardiovascular responses to glutamate microinjection in the dorsomedial periaqueductal gray of unanesthetized rats. J Neurosci Res. 2012;90:2193‐2200. 22715034 10.1002/jnr.23094

[58] Ishide T , Hara Y , Maher TJ , Ally A . Glutamate neurotransmission and nitric oxide interaction within the ventrolateral medulla during cardiovascular responses to muscle contraction. Brain Res. 2000;874:107‐115. 10960594 10.1016/s0006-8993(00)02562-2

[59] Sanada S , Node K , Minamino T , et al. Long‐acting Ca2+ blockers prevent myocardial remodeling induced by chronic NO inhibition in rats. Hypertension. 2003;41:963‐967. 12629037 10.1161/01.HYP.0000062881.36813.7A

[60] Resstel LBM , Corrêa FMDA . Medial prefrontal cortex NMDA receptors and nitric oxide modulate the parasympathetic component of the baroreflex. Eur J Neurosci. 2006;23:481‐488. 16420454 10.1111/j.1460-9568.2005.04566.x

